# A Randomized Trial of a Swimming-Based Alternative Treatment for Children with Attention Deficit Hyperactivity Disorder

**DOI:** 10.3390/ijerph192316238

**Published:** 2022-12-04

**Authors:** Soukaina Hattabi, Pedro Forte, Filip Kukic, Asma Bouden, Mona Have, Hamdi Chtourou, Andrew Sortwell

**Affiliations:** 1 High Institute of Sports and Physical Education of Elkef, University of Jendouba, El Kef 7100, Tunisia; 2 Research Unit UR12SP20, Razi Hospital, Mannouba 2010, Tunisia; 3 Department of Sports, Higher Institute of Educational Sciences of the Douro, 4560-708 Penafiel, Portugal; 4 Department of Sports Sciences, Instituto Politécnico de Bragança, 5300-253 Bragança, Portugal; 5 Research Center in Sports, Health and Human Development, 5001-801 Vila Real, Portugal; 6 Abu Dhabi Police, Abu Dhabi 253, United Arab Emirates; 7 Department of Child Psychiatry, Razi Hospital, Tunisia Faculty of Medicine of Tunis, Manouba 2010, Tunisia; 8 Centre of Research in Childhood Health, Department of Sports Science and Clinical Biomechanics, University of Southern, Campusvej, 55, 5230 Odense, Denmark; 9 Higher Institute of Sport and Physical Education of Sfax, Sfax 3000, Tunisia; 10 School of Nursing, Midwifery, Health Sciences and Physiotherapy, University of Notre Dame Australia, Sydney 2007, Australia

**Keywords:** adapted aquatic activity, motor control, disruptive behavior, academic performance

## Abstract

Attention deficit hyperactivity disorder (ADHD) is considered a highly prevalent neurodevelopmental childhood disorder extending from ages 1–2 to 12–13, associated with impairment across multiple domains, including social, emotional, and cognitive functioning. Little is known about alternative treatments for this disorder. Interest has grown in physical activity as a potential intervention for rehabilitating children with ADHD. This study aimed to investigate the impact of adapted swimming activity on cognitive functions, academic performance, and related behavior of Tunisian children with ADHD. The study was conducted on school children aged 9 to 12 years (*n* = 40, 5 female and 35 male) diagnosed with ADHD. They were randomly assigned to an experimental group (exercise intervention) or the control group. The Hayling test was used to assess cognitive performance, the Children Behavior Check List (CBCL) was used to assess ADHD-related behavior, and the change in reading and numeracy proficiency was assessed pre- and post-intervention. After 12 weeks of the intervention, the results revealed that there were significant improvements in behavior (*p* < 0.001), inhibition process (*p* < 0.001), and academic performance (*p* < 0.001) in the experimental group compared with the control group. These findings suggest that adapted swimming activity may have positive implications for cognitive function, behavior, and academic performance. This research may provide preliminary support for alternative therapeutic interventions that could be used by practitioners. Moreover, the results support active practice of recreational physical activities as a strategy to support children in overcoming ADHD deficiencies.

## 1. Introduction

Attention deficit hyperactivity disorder (ADHD) is considered the most frequent neurodevelopmental disorder in children [1,2]. ADHD is characterized by three core attributes: inattention, hyperactivity, and impulsivity [3,4,5,6]. These attributes usually appear before the age of seven and can create challenges and difficulties in different social settings, such as school, home, or recreational activities [7], resulting in cognitive impairments such as: inhibitory control [8], time perception [9], memory [10], and attention deviation [11]. A meta-regression analysis of 102 epidemiological studies showed that ADHD has a global prevalence of 5.29% [12], whereby the incidence in boys was three times higher than in girls (9.2% vs. 3.0%) [13,14,15]. Genetics researchers believe that altered dopaminergic functions play a crucial role because they do not adequately modulate non-dopaminergic signaling. The malfunctioning of the dopaminergic system occurs at three levels [16,17]. The first level includes the hypofunction of the mesolimbic dopamine branch that provokes the alteration of the reinforcement process and the extinction of operative responses [17]. This results in a lack of sustained attention, hyperactivity, excessive behavioral variability, and motor and cognitive impulsiveness. The second level includes hypo-functioning of the nigrostriatal dopamine branch that provokes weak motor control [18]. Finally, the third level includes hypo-functioning of the mesocortical branch that causes weakness in non-declarative learning [19]. The under-functioning dopamine branch results in the dominant individual tendency in children with ADHD to be less enthusiastic about reinforcement [20,21]. Therefore, under normal circumstances, they do not have sufficient motivation to function at a typical child’s level [20,22,23]. Thus, the theory predicts that interactions between individual predispositions and the social environment lead to ADHD behaviors and symptoms. Hence, the approach proposes that ADHD symptomatology results in the interplay between individual susceptibility and the social environment. The ADHD disorder may vary depending on factors that positively and negatively affect their development, defining particular requirements for appropriate parenting and societal approaches [24]. Therefore, by minimizing the ADHD symptoms, children may build more positive experiences and interactions with their peers [25], resulting in increased social inclusion. It has recently been reported that individual predispositions to the disorder may interact with optimal parenting and societal styles and produce stable behavioral patterns [16,17,26]. Therefore, it is important to explore interventions that may alleviate the symptoms of ADHD.

Managing cognitive, behavioral, and emotional impairment associated with ADHD can include drug treatment, non-pharmaceutical interventions, and multiple therapeutic modalities [27]. Despite the pharmacological treatment that has demonstrated varying levels of effectiveness for many years, it is not the preferred first line of therapy [27]. Alternatives to pharmacological treatments, such as physical exercise, have emerged to support children with ADHD to manage the impairments [28]. Physical activities have been reported to induce positive effects that can be classified into the following categories: physical [29], behavioral [30], cognitive [31], emotional, spiritual, and social health [32]. The cognitive health benefits associated with physical activity are well-established as principal means of intervention [33]. For instance, recreational swimming games have been found to enhance cognitive processes and reduce behavioral and emotional dysfunctions. These results from the exercise have a positive effect on the brain’s structural growth, functional neurocognitive development, and the long-term development trajectory of ADHD [34]. Swimming lessons’ importance has increased worldwide regarding children’s recreational activities [35,36]. Additionally, swimming allows for the promotion of age-appropriate motor and physical experiences contributing to the children’s development [37]. Furthermore, the physical and physiological effects of swimming may also contribute to developing motor performance skills [38] and efficient movement patterns, leading to overall motor advancement. Additionally, children between 5 and 10 years old are prone to have a higher gross motor efficiency in comparison to non-swimmers [39]. A previous study has proven that recreational swimming programs in children with ADHD (aged between nine and twelve years old) improved memory accuracy, selective attention and inhibition processes [39,40], and mental health in children between 11 and 14 years old [41,42]. This may suggest swimming as an effective therapy for children with ADHD. However, there is a research gap about the effects of recreational swimming activity on cognitive functions, academic performance, and disruptive behavior, especially in Tunisian children.

Regarding the above, the goal of this research was to investigate the impact of a recreational swimming program on the symptomatology of children with ADHD aged nine to twelve years and the effects on cognitive functions, academic performance, and disruptive behavior. Accordingly, this study hypothesized that a recreational swimming activity program would positively affect cognitive functions, academic performance, and disruptive behavior of a sample of Tunisian children with ADHD.

## 2. Materials and Methods

### 2.1. Study Design

The study was a 12-week school-based, cluster-randomized controlled trial. Schools were the cluster unit for randomization. The research was conducted with primary school students from ten schools (five interventions, five controls) in Tunisia. Each school was randomly allocated to either ‘control’ or ‘intervention’ status. The randomization was performed by an external researcher who did not partake in other parts of the study. The study was approved by Sfax University Research Ethics Committee (ref: CPP-02/18). A 12-week recreational swimming program was applied, and its effects on baseline measures were tested and analyzed.

### 2.2. Participants

The study participants were 40 children aged from 9 to 12 years with ADHD with varying levels of severity (see Table 1). Participants were recruited from 10 Tunisian primary schools using the Conner’s Scale, which was translated and validated in Arabic [41]. Before selection, a clinical interview was conducted by a psychiatrist with potential participants. The interview was supplemented with a recognized standardized assessment known as a semi-structured questionnaire, the Kiddie-Schedule of Affective Disorders and Schizophrenia Present and Lifetime Version (K-SADS-PL, version 1.0, 1996). Participants were also required to pass a proof of development quotient colored progressive matrix (QD) [43]. They were not mentally or physically delayed and were right-handed. The study followed the Helsinki Declaration [44]. Parents were provided with the study protocol and written consent. Parents or guardians gave written informed consent allowing their child to participate. The parents or adolescents could revoke this consent at any time.

### 2.3. Intervention Training Program

The swimming training sessions were led by three professional coaches with experience in water exercise. The swimming program (see Table 2) duration was 12 consecutive weeks, with 3 sessions per week. The program was implemented in a 20 m swimming pool at the High Institute of Sport and Physical Education of El Kef, and the pool temperature was 29 ± 1 °C. It was held on Monday, Wednesday, and Friday from 9:00 a.m. to 12:00 p.m. to avoid the circadian rhythm problem. Each session lasted 90 min and was divided into three stages: a 15 min warm-up, 70 min of aquatic exercises, and a 5 min cool-down, as prescribed [41,42]. The focus of the swimming lessons during the first four weeks was to promote the students’ familiarization with the aquatic environment and develop basic aquatic skills. In the next four weeks, aquatic specialists focused on the development of specific skills and analytics swimming. In the last four weeks, students focused on specific motor efficiency by practicing as much movement as possible through repetition of some tasks but also using variations in the exercise forms.

The intensity of training was assessed using heart rate (HR) monitors. Within this study, the target heart rate was 50–70% of the maximum HR using the Karvonen equation: (Maximum HR − Resting HR) × (50–70%) + Resting HR [45]. This was then adjusted for water by subtracting 15 heartbeats, so that the target HR ranged from 135 to 160 beats per minute [45].

### 2.4. Measure of Outcomes

All measurements were obtained twice, first at baseline and then at the end of the intervention. The test procedures were the same at both time points and conducted by the same research assistants. The following physiological measures were assessed: anthropometrics (body weight and height), resting HR, maximum HR, and peak oxygen consumption (VO_2_ peak (mL/kg/min)) to assess participants’ physical fitness (20 m shuttle run test). Inhibitory control was also assessed using the Junior Hayling test [46]. Behavioral and emotional processes were evaluated using the Child Behavior Checklist (CBCL), while academic performance was evaluated using assigned reading comprehension, math, and final grade point average grades.

#### 2.4.1. The Junior Hayling Test

The Junior Hayling test [47] was divided into “A” and “B” sections. Each section was made up of ten sentences, with the final word omitted. The word that completes the sentence in part “A” (initiation) must fit appropriately at the end of it. Part “B” (inhibition) required the child to complete the sentence with a word that makes no sense in the context of the sentence; if a child provided an answer that gives meaning to the phrase, the task instructions are repeated. We moved on to the next sentence if the latency exceeded 60 s. The response time (in seconds) and the words produced were both recorded in both parts. We calculated the response time as the difference between the latencies in parts “A” and “B” (B − A). Each response in part “B” for the produced words was scored based on its semantic similarity to the stimulus sentence. A one-point penalty was applied if the child produced a word that was semantically related to the sentence, and a three-point penalty was applied if the child produced a word that was simply a sentence completion [48]. In both parts, the response time (in seconds) and the produced word were recorded. For the response time, the “Additional Thinking Time” (ATT) was calculated as the difference between the latencies in parts “A” and “B” (B − A).

#### 2.4.2. The Child Behavior Checklist (CBCL)

The Child Behavior Checklist for 4–18 years old (CBCL/4–18), Arabic form, was reported by parents, and validated by [49]. It was created to assess children’s abilities and problems [50]. It is an important tool in the assessment of the emotional, behavioral, and social needs of children and youth [51]. Achenbach and Ruffle included two components (competencies and problems) to better understand a particular child’s mental health status [52]. Although both sections seem complementary, we included only the behavioral problems section in our study because parents had filled it in. The profile displays a child’s standing on syndromes of problems. Each syndrome consists of problems that were found to occur concomitantly [50]. Syndromes of the CBCL/4–18 are social problems, attention/hyperactivity problems, delinquent behavior, aggressive behavior, withdrawn somatic complaint, anxious/depression, and thought problems. We can consider the subscale of attention hyperactivity items as tools of screening the diagnostic of ADHD impairment. It took about 10 min for parents to fill in the form [52], while for the researcher to score it by hand on the child’s profile, it took another 10–15 min.

#### 2.4.3. Academic Performance

The situation of the school status makes it possible to identify the “ranking” of the performances of a given child in the group to which he belongs. The overall pedagogical evaluation of the child is based on the curricula and on the results that a child obtained in relation to a given “grade”. The most traditional classifications (3 levels) concern the “low”, “medium”, or “high” levels. Academic performance was evaluated by using grades obtained in the exams of reading comprehension, math, and overall average before and after the physical activity intervention.

### 2.5. Statistical Analysis

All statistical analyses were performed using SPSS (version 21.0) for Windows (SPSS Inc., Chicago, IL, USA). A significance level of 0.5 was used prior to the adjustment. For this study, examination of the normal distribution by the Kolmogorov–Smirnov test revealed that the indices coming from Hayling tasks, behavioral/comorbidities assessment, and academic performance were normally distributed. Independent *t*-tests and Student’s *t*-test for matched samples were performed to compare the demographic and physical variables and cognitive tasks between the aquatic exercise group and the control group and between the same group before and after the intervention. In addition to the test exercise interaction manipulation, a mixed 2 (group: exercise vs. control) × 2 (time: pre and post) analysis of variance (ANOVA) was completed. A ἠ^2^ (eta square) value for the ANOVAs was used as an index of the effect size. The interpretation of ἠ^2^ was as follows: 0 < η^2^ ≤ 0.04, minimum; if 0.04 < η^2^ ≤ 0.25, moderate; if 0.25 < η^2^ ≤ 0.64, strong; if η^2^ > 0.64, large effect [53]. If a significant repeated measures ANOVA was calculated (*p* < 0.05), a post hoc pairwise comparison (Bonferroni adjusted) was completed. The use of the follow-up pairwise comparison was used to examine the location of the significant difference, whether between-subject and/or within-group.

## 3. Results

The assumptions of normality and homogeneity were met for all included data (*p* > 0.05). The descriptive statistics and comparative analysis (Student’s *t*-test) between the experimental group and the control group for baseline values revealed that there was only one statistical difference between groups (Table 3). Table 3 shows the comparison of group changes for all tests.

### 3.1. Maximum Oxygen Consumption

For the aerobic capacity incremental running test, Luc Léger’s Shuttle test was adopted to determine the MAV and to estimate the VO_2_max. It was performed on a 20 m running track bounded by two cones. Each participant began with a running speed of 8.5 km/h between two lines (20 m apart), with consecutive speed increases of 0.5 km/h every minute until exhaustion. Each participant must adjust their running velocity to reach the correspondent cone, concomitantly with the soundtrack signal. The test ended when the subject could no longer maintain the required speed imposed by the beep, or when they were not able to reach the following cone in due time. During the Luc Léger test, HR was recorded using an HR monitor (810, Polar, Kempele, Finland).

For maximum oxygen consumption, the repeated measures ANOVA results indicated that after the intervention, only maximum oxygen consumption (*F* (1, 38) = 102.001, *p* < 0.001, ἠ^2^ = 0.729) increased, with a large effect in the experimental group. Bonferroni-adjusted post hoc pairwise comparisons indicated no significant difference between maximum oxygen consumption scores for the experimental and control groups pre-intervention (*p* = 1.000), but significantly higher maximum oxygen consumption scores for the experimental group compared to the control group post-intervention (*p* ≤ 0.001) (see Table 3).

### 3.2. Hayling Test—Analysis of Temporal Data

**Figure 1:**
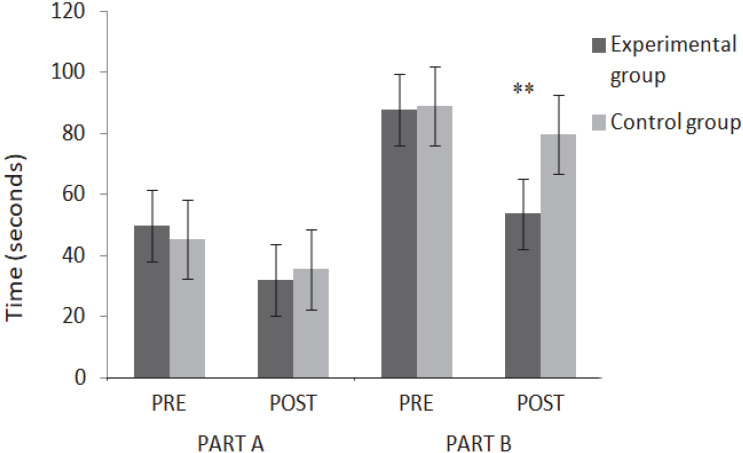
Mean Hayling test completion times for the two groups before and after the intervention. ** *p* < 0.001.

The ANOVA showed a significant interaction of the factors “group × evaluations” for the latency time of part A (F (1, 38) = 8716, *p* = 0.005, ἠ^2^ = 0.187), with a large effect, and part B (F (1, 38) = 19,205, *p* < 0.001, ἠ^2^ = 0.336) with a large effect. Three months of swimming training based on games led to a significant improvement in the execution time of part A (−32.34%, *p* < 0.001) and part B (−41.66%, *p* < 0.001) for the experimental group. On the other hand, no significant changes occurred in the control group (−12.44%, *p* > 0.05 and −11.218%, *p* > 0.05, respectively). The comparison between the two groups showed a very significant difference in part B latency time that requires inhibition of a dominant response only after 12 weeks (see Figure 1).

**Figure 2:**
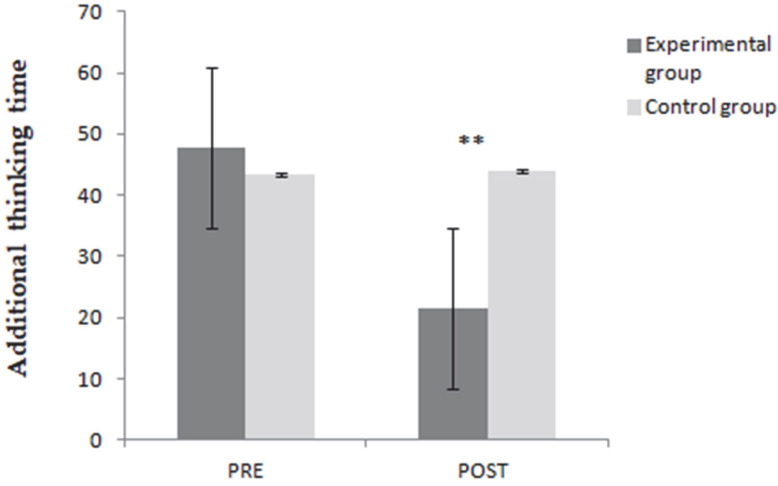
Pre- and post-intervention additional thinking time scores for the two groups. ** *p* < 0.001.

In addition, ATT revealed a significant effect of the training program in favor of the experimental group with (F (1, 38) = 16,286, *p* ˂ 0.001, ἠ^2^ = 0.300), which is presented in Figure 2. Bonferroni-adjusted post hoc pairwise comparisons indicated no significant difference between ATT scores for the experimental and control groups pre-intervention (*p* = 0.56), but significantly different ATT scores for the experimental group compared to the control group post-intervention (*p* ≤ 0.001).

Regarding the error score, the two groups had equivalent error scores (F (1, 38) = 0.081, *p* ˃ 0.05, ἠ^2^ = 0.002) with minimal effects in the automatic condition (part A) before and after the training period. However, a significant effect of training occurred in the experimental group in part B of the test (F (1, 38) = 78.114, *p* ˂ 0.001, ἠ^2^ = 0.673), with a large effect.

### 3.3. CBCL Tasks

Comparisons between the experimental and control groups on pre-test measures did not reveal any statistically significant differences in CBCL tasks (Table 3). However, after the exercise intervention, parents of the children in the experimental group reported lower CBCL scores compared to scores reported by parents of children in the control group. Starting with the externalizing behavior items, the interaction between the group variable and the time variable (pre- and post-program) was in favor of the experimental group, with a large effect (F (1, 38) = 375.317, *p* < 0.0001, ἠ^2^ = 0.908). Children from the experimental group had significantly lower social problems, with a large effect (F (1, 38) = 153.005, *p* < 0.0001, ἠ^2^ = 0.801), attention problems (F (1, 38) = 239,121, *p* = 0,000, ἠ^2^ = 0.863), delinquent behavior with a large effect (F (1, 38) = 181.809, *p* < 0.0001, ἠ^2^ = 0.827), and aggressive behavior with a large effect (F (1, 38) = 278.517, *p* < 0.0001, ἠ^2^ = 0.880). Bonferroni-adjusted post hoc pairwise comparisons indicated no significant difference between all CBCL measures for the experimental and control groups pre-intervention in externalizing (*p* = 0.067), internalizing (*p* = 0.98), aggressive (*p* = 0.273), delinquent (*p* = 0.51), attention (*p* = 0.246), and social (*p* = 0.388) behaviors, but post-intervention the experimental group had significantly higher externalizing behavior (*p* ≤ 0.001), lower social problems (*p* ≤ 0.001), lower attention problems (*p* ≤ 0.001), reduced delinquent behaviors, reduced aggressive behavior (*p* ≤ 0.001), and maximum oxygen consumption scores for the experimental group compared to the control group post-intervention (*p* ≤ 0.001).

Concerning internalizing behavior items (Table 4), the interaction also revealed a lower score of all items after the exercise intervention. Statistical analysis showed a tendency for a significant reduction in somatic complaints, anxious/depressed, thought problems, and feeling withdrawn problems. The Bonferroni-adjusted post hoc pairwise comparisons indicated no significant differences between internalizing measures for the experimental and control groups pre-intervention for feeling withdrawn (*p* = 1.000), thought (*p* = 0.250), anxiety/depressed (*p* = 0.860), somatic (*p* = 1.000), and externalizing problems. Post-intervention, the experimental group had significant improvement in somatic complaints (*p* < 0.001), feeling anxious/depressed (*p* < 0.001), thought problems (*p* < 0.001), and withdrawn problems (*p* < 0.001).

### 3.4. Academic Performance

No significant differences were found at the pre-test between the two groups for academic achievement (Table 5). Following the training program, the analysis revealed that significant post-test differences were observed for academic performance. The experimental group exhibited enhanced performance after exercise on marks of reading comprehension with a strong effect, math with a strong effect, and finally, better pass marks with a strong effect. Bonferroni-adjusted post hoc pairwise comparisons indicated no significant differences between reading comprehension scores for the experimental and control groups pre-intervention (*p* = 0.830), but significantly higher scores for the experimental group compared to the control group post-intervention (*p* < 0.001). Similarly, Bonferroni-adjusted post hoc pairwise comparisons indicated no significant differences between math scores for the experimental and control groups pre-intervention (*p* = 0.890), but significantly higher scores for the experimental group compared to the control group post-intervention (*p* ≤ 0.001). For the pass mark, Bonferroni-adjusted post hoc pairwise comparisons indicated no significant difference between reading comprehension scores for the experimental and control groups pre-intervention (*p* = 0.780), but significantly higher scores for the experimental group compared to the control group post-intervention (*p* < 0.001).

## 4. Discussion

This study investigated the effects of 12-week recreational swimming activity on cognitive tasks, disruptive behavior, and academic performance in ADHD youth. According to the results, the applied exercise program improved the cognition and behavior of ADHD children, as reflected by the experimental group showing better results in CBCL and Hayling tests. In addition, children from the experimental group showed improved academic performance in reading comprehension and math. Therefore, organized recreational swimming activities could be considered an effective approach to improving the performance of ADHD children. Our findings are consistent with previous research that found a physical activity program beneficial for response inhibition in children with ADHD [5,41,42].

In the present study, an indirect valid and precise dry land test was used to assess swimmers’ physiological capacity (VO_2_ max) [54]. Typically, swimmers’ oxygen consumption is assessed post-swim and with direct (oximetry) and indirect (experimental or analytical estimations) tests [55]. In the present research, the intervention group practiced swimming lessons and the control group did not enroll in aquatic/swimming activities. Thus, the peak oxygen consumption (VO_2_ peak (mL/kg/min)) was used to assess participants’ baseline physical fitness (20 m shuttle run test). This test allowed comparing the oxygen consumption between the two groups. In the present study, physiological gains were obtained in the intervention group as expected and supported by the literature [39]. Smith and colleagues [56] found that an 8-week physical exercise program improved inhibitory control in 14 children at risk for ADHD (30 min, 5 times a week). Research by Hung et al. [57] and Piek et al. [58] showed an association between improved motor skills and cognitive functions, particularly when the exercise sessions were designed to improve participants’ attention and concentration. Accordingly, swimming games may have a positive effect on aspects of neurocognitive function and inhibitory control in ADHD children. Swimming allows to improve motor skills, physical fitness, cognition, and social skills [59]. This can be explained by the structure and the regiment of swimming lessons, where the pace and velocity are controlled by the swimmer and allow to self-define a racing strategy, related to decision-making in a specific environment [59]. In another investigation into the effect of physical fitness training and academic achievement in sixth- and seventh-grade students, aerobic fitness and behavior test scores were related to reading and mathematics achievement [60]. In accordance with previous studies, the current study provides further support to enhanced academic performance resulting from the benefits of exercise, such as improved attention and concentration. However, the improvements in math are somehow expected. Children without any disorder are more prone to improve math performance with physical activity and exercise [61,62]. This is because, as the motor skills and motor proficiency improve, so do the cognitive skills and academic performance in math. The authors of [42] assessed the effect of swimming training on mental health parameters, cognition, and motor coordination in children with ADHD. The training group showed that the aquatic exercise program improved parameters related to depression, stress, cognition, selective attention, lower limbs’ coordination and laterality, flexibility, and abdominal resistance. These findings support the results of the present research. Additionally, physical education [63], physical activity, and exercise [64,65,66] improve muscular and cognitive function, which are related to academic performance. Finally, Sabzi et al. [67], in a randomized control trial with 23 children, reported that a water treadmill exercise program reduced the levels of behavior, social, and psychosomatic problems, anxiety/shyness, and the overall score of ADHD [67].

Games could be an effective way to effectively enhance performance in ADHD [7], and practitioners of a variety of theoretical persuasions have been using games while working with children. Mitigating the effects of exercise on ADHD dysfunction that are related to inhibitory control could be explained by neuroelectric adjustment. For instance, neuroelectric adjustment could explain the mitigating effects of exercise on ADHD dysfunctions that are related to inhibitory control. Moreover, Hillman et al. [68] investigated the acute effects of aerobic exercise on cognition and discovered that participants had larger P3 amplitudes and shorter P3 latency. Since the amplitude and latency of P3 reflect the amount of attention and the speed of stimulus evaluation and recognition processes, acute exercise could be beneficial in improving neuroelectric activity. In the current study, ADHD patients exhibited dysfunction in cerebral zones such as the dorsolateral prefrontal cortex and the orbitofrontal cortex of the prefrontal cortex [69]. These zones are essential for executive functioning, cognitive process control, and decision-making [70]. Dopamine release, which has been linked to exercise [71], influences the functioning of these zones, which is another mechanism that may contribute to inhibitory control in children with ADHD [72]. Finally, in this present study, aquatic swimming games were adopted in the experimental group’s swimming lessons. Games with creative and playful methodologies allow to motivate children to participate in physical activities [73,74,75]. This may also sustain and explain the positive results of this intervention.

Education systems and schools can promote, within their communities, the benefits of frequent swimming sessions for students with ADHD, especially considering all the associated health benefits compared to the use of medications. Based on the findings of this study, three 90-min sessions a week of low aerobic intensity swimming can be advised as a strategy to support students whose ADHD condition is negatively impacting on learning and engagement in classes.

## 5. Limitations

There are some constraints that must be addressed. Firstly, our sample size was small; however, the sample size was similar to previous studies [42]. One of the most challenging aspects of the study was that the sample recruitment and diagnosis was highly advanced. In future studies, the process for recruitment of study participants needs to be improved to achieve more significant and generative results in children with ADHD. Secondly, because this study only includes children with ADHD, it is recommended that the effect of the exercise intervention on ADHD be investigated in comparison to typically developing children. Finally, while this study included male and female ADHD children, the effect of the intervention on gender was not investigated. Previous research has shown that gender differences significantly affect cognitive tasks after exercise. As a result, it will be critical to investigate the effect of gender differences on cognitive, behavioral, and emotional functions in ADHD children.

## 6. Conclusions

Attention deficit hyperactivity disorder (ADHD) causes impairment in a variety of areas, including social, familial, emotional, and academic functioning. Alternative treatments for this disorder are poorly understood. As a result, greater interest in physical activity as a potential intervention for rehabilitating children with ADHD has increased. In conclusion, the results of the present study have important practical implications. Our findings showed that a 12-week recreational swimming program positively affected the behavioral, cognitive, and academic performance of children with ADHD. The findings suggested that recreational swimming exercise could be an effective tool for working with children with ADHD to improve their functioning and academic performance. The training protocol of this study may be used by physical education teachers as well as sports medicine professionals. This could be a non-pharmacological treatment for ADHD and preliminary support for therapeutic interventions.

## Figures and Tables

**Table 1:** Baseline characteristics of the experimental and control groups.

Variables	Experimental Group (*n* = 20)	Control Group (*n* = 20)
Gender (Females/Males)	3/17	2/18
Age (years)	9.95 ± 1.31	9.75 ± 1.33
Weight (kg)	34.95 ± 8.95	34.70 ± 6.6
Height (m)	1.4 ± 1	1.4 ± 0.73
BMI (kg/m^2^)	17.56 ± 2.91	17.54 ± 2.31
VO_2_max (mL/L/min)	35.35 ± 2.09	35.79 ± 2.16
Resting HR (bpm)	74.85 ± 4.09	74.85 ± 4.09
ADHD-I	4 (20%)	5 (25%)
ADHD-HI	6 (30%)	4 (20%)
ADHD-C	10 (50%)	11 (55%)

Note: BMI = body mass index; ADHD-I = inattentive subtype; ADHD-HI = hyperactive-impulsive subtype; ADHD-C = combined hyperactive-impulsive and inattentive subtype. Resting HR: resting heart rate.

**Table 2:** Intervention training program: typical swimming session.

Exercise Category	Activities
Warm-up period	Duration: 15 min. Intensity: (moderate intensity) <50% of maximum HR. The noodle attack: Children stand in a circle in a shallow area of the pool and hold hands. One child is in the center of the circle, and spins noodle gently on the surface of the water like a clock. The children must avoid being hit by the fry by hiding under the water. The one who is hit replaces the one in the middle.
Aerobic conditioning games	Duration: 70 min. Intensity: 50–70% of maximum HR (vigorous intensity). Swimming laps freestyle, backstroke, elementary backstroke, breaststroke, and only legs kicking while using a kickboard. Movement activities while standing in the shallow and running in place, jumping jacks, reciprocal arm and leg movements, hopping on one foot, jumping in place, and jumping forwards, backwards, and sideways Relay races in the shallow end running from one side of the pool to the other: (1) filling buckets with balls or other pool toys, (2) shooting a ball into a hoop, or (3) running in teams while holding onto the aquatic noodles. Obstacle courses: running in the water or swimming while going under, over, and around obstacles or retrieving dive rings. Games: playing ball by rapidly catching, throwing, and shooting baskets into a basketball net, playing keep the ball away from the coaches, and straddle sitting on the aquatic noodle and ‘‘racing the horse’’ the length of the pool
Cool-down and flexibility	Duration: 5 min. Intensity: stretches are held for 20–30 s and repeated twice for each side, <50% of maximum HR. Movement activities in the water include marching in place, and arm and leg circles. Performed at a slow pace to bring HR down to lower than the target range. Gently stretching of pectorals, latissimus, triceps, hamstring, quadriceps, plantar flexors, and lateral flexion trunk stretch. Performed in the shallow end of the pool using the pool wall for balance as needed.

**Table 3:** Baseline homogeneity between groups for variables.

Variable	Experimental Group M ± SD	Control Group M ± SD	*p*-Values
Age	9.95 ± 1.31	9.75 ± 1.33	0.70
Height	1.4 ± 1.0	1.4 ± 0.73	0.15
Weight	34.95 ± 8.95	34.70 ± 6.60	0.36
BMI	17.56 ± 2.91	17.54 ± 2.31	0.36
Part A Hayling test	49.85 ± 18.97	45.40 ± 16.87	0.44
Part B Hayling test	97.69 ± 41.14	88.55 ± 27.72	0.42
EsB	22.50 ± 3.72	20.35 ± 3.03	0.05
Weight (kg)	34.95 ± 8.95	34.70 ± 6.60	0.92
VO_2_ peak	35.35 ± 2.09	35.78 ± 2.16	0.59
Reading marks	11.45 ± 1.46	11.55 ± 1.43	0.83
Math	5.95 ± 2.16	6.05 ± 2.11	0.88
Pass marks	10.057 ± 0.99	10.14 ± 0.92	0.78
Resting HR (bpm)	74.85 ± 4.09	74.85 ± 4.09	1.00
Internalizing behavior	19.15 ± 6.49	19.20 ± 7.05	0.98
Externalizing behavior	26.10 ± 2.86	24.65 ± 1.90	0.07
Aggressive behavior	17.10 ± 1.55	16.55 ± 1.57	0.27
Delinquent behavior	9.00 ± 1.86	8.10 ± 0.72	0.06
Anxiety/depression	7.35 ± 2.58	7.50 ± 2.68	0.86

Note: EsB: error score of part B.

**Table 4:** Mean, standard deviation, and ES for the CBCL test.

Variables	Experimental Group (*n* = 20)		Control Group (*n* = 20)		Effect Size	Level of Effect Size
	Pre	Post	Pre	Post		
Withdrawn	5.50 ± 1.82	3.85 ± 0.59	5.40 ± 2.09	5.75 ± 2.00	0.283 **	Strong
Somatic complaints	6.30 ± 2.32	4.20 ± 0.83	6.30 ± 2.47	6.65 ± 2.50	0.303 **	Strong
Anxious/depressed	7.35 ± 2.58	4.50 ± 0.95	7.50 ± 2.69	7.60 ± 2.89	0.307 **	Strong
Thought problems	6.80 ± 1.44	4.55 ± 0.76	7.45 ± 2.01	7.55 ± 1.90	0.481 **	Strong
Social problems	10.70 ± 0.80	6.80 ± 1.24	10.45 ± 1.00	10.50 ± 0.95	0.801 **	Large
Attention problems	15.10 ± 0.85	9.35 ± 0.93	14.75 ± 1.02	14.55 ± 1.23	0.863 **	Large
Delinquent behavior	9.00 ± 1.86	4.90 ± 1.65	8.10 ± 0.72	8.35 ± 0.81	0.827 **	Large
Aggressive behavior	17.10 ± 1.55	9.75 ± 1.45	16.55 ± 1.57	16.35 ± 1.09	0.880 **	Large
Internalizing behavior	17.10 ± 1.55	12.55 ± 2.04	19.20 ± 7.05	20.00 ± 7.14	0.324 **	Strong
Externalizing behavior	26.10 ± 2.86	14.65 ± 2.85	24.65 ± 1.90	24.70 ± 1.38	0.908 **	Large

Note: ** Significant at *p* < 0.001; ES: effect size.

**Table 5:** Mean, standard deviation, and effect size for the academic performance.

Variables	Experimental Group (*n* = 20)		Control Group (*n* = 20)		Effect Size	Level of Effect Size
	Pre	Post	Pre	Post		
Reading	11.45 ± 1.47	13.80 ± 1.36	11.55 ± 1.43	11.25 ± 1.16	0.368 **	Strong
Math	5.95 ± 2.16	9.20 ± 1.57	6.05 ± 2.11	5.85 ± 1.46	0.595 **	Strong
Pass mark	10.06 ± 1.00	11.70 ± 0.96	10.14 ± 0.91	10.18 ± 0.91	0.396 **	Strong

Note: ** Significant at *p* < 0.001.

## Data Availability

Not applicable.
